# The Role of MicroRNA in the Airway Surface Liquid Homeostasis

**DOI:** 10.3390/ijms21113848

**Published:** 2020-05-28

**Authors:** Nilay Mitash, Joshua E. Donovan, Agnieszka Swiatecka-Urban

**Affiliations:** Department of Pediatrics, UPMC Children’s Hospital of Pittsburgh, University of Pittsburgh School of Medicine, Pittsburgh, PA 15224, USA; jed123@pitt.edu

**Keywords:** microRNA, airway surface liquid, miRNA-mRNA interaction, airway host defense, ion channels, RNA-induced silencing complex, cystic fibrosis, chronic obstructive pulmonary disease, coronavirus, SARS-CoV-2

## Abstract

Mucociliary clearance, mediated by a coordinated function of cilia bathing in the airway surface liquid (ASL) on the surface of airway epithelium, protects the host from inhaled pathogens and is an essential component of the innate immunity. ASL is composed of the superficial mucus layer and the deeper periciliary liquid. Ion channels, transporters, and pumps coordinate the transcellular and paracellular movement of ions and water to maintain the ASL volume and mucus hydration. microRNA (miRNA) is a class of non-coding, short single-stranded RNA regulating gene expression by post-transcriptional mechanisms. miRNAs have been increasingly recognized as essential regulators of ion channels and transporters responsible for ASL homeostasis. miRNAs also influence the airway host defense. We summarize the most up-to-date information on the role of miRNAs in ASL homeostasis and host–pathogen interactions in the airway and discuss concepts for miRNA-directed therapy.

## 1. Introduction

A variety of airborne pathogens and abiotic environmental particles can enter the airspace during inhalation. The host protects the airway integrity by a multi-layered defense mechanism directed at eliminating the unwanted particles. A coordinated function of different airway epithelial cells, such as multi-ciliated, club, serous, goblet, ionocytes, the resident macrophages, the host immune system, and the airway surface liquid (ASL) coating the luminal surface of the airway epithelium, shapes the airway host defense.

## 2. ASL Homeostasis During Health and Disease 

Two distinct layers, the superficial mucus and the deeper periciliary liquid [1], comprise the ASL in the trachea and bronchi [1]. The submucosal glands and goblet cells secrete mucus, which traps inhaled pathogens. The aqueous periciliary layer, secreted by serous cells, allows cilia to perform the periciliary clearance [2]. The volume of the periciliary layer and mucus hydration is regulated by the transcellular and paracellular movement of ions and water [3]. Chloride (Cl^−^) and sodium (Na^+^) are the primary ions involved in the ASL homeostasis, and both are present at ~100-130 mM concentration. Potassium (K^+^) and bicarbonate (HCO3^−^) are also relevant but exist at much lower concentrations (20 mM and 10 mM, respectively). The electrochemical gradient determines the airway epithelial ion transport. Cl^−^, taken up by the cells via the basolateral Na+/K+/2Cl^−^ co-transporter, is secreted apically by the Cystic Fibrosis Transmembrane Conductance Regulator (CFTR) and Calcium (Ca^+2^)-activated Cl^−^ Channels (CaCCs), such as Anoctamin-1 (ANO1), also known as Transmembrane member 16A (TMEM16A). Na^+^ and K^+^ exit the cell via the basolateral Na^+^/K^+^-ATPase, and K^+^ is recycled via the basolateral K^+^ channels. Na^+^ is absorbed apically via the Epithelial Na^+^ Channel (ENaC). During Cl^−^ secretion, Na^+^ and water move paracellularly; hence, the ASL volume increases, but ion concentration remains unchanged. K^+^ secretion through the apical big K^+^ (BK) large conductance, Ca^+2^-activated, and voltage-dependent K^+^ channel facilitates Cl^−^ efflux by hyperpolarizing the apical membrane and increasing the force for Cl^−^ secretion by acting as a counter-ion. The solute carrier family 26 member A9 (SLC26A9) is an epithelial anion transporter expressed in the airway that functions as a Cl^−^ channel with minimal conductance to HCO3^−^ and contributes to Cl^−^ secretion [4,5]. SLC26A4 and non-gastric H+/K+-ATPase (ATP4B) are also expressed and may also contribute to the ASL homeostasis. Ion channel defects that compromise ASL homeostasis impair mucociliary clearance and lead to ASL dehydration, airway obstruction with mucus, respiratory infections, and progressive decrease in the lung function. This sequence of events results from mutations in the *CFTR* gene and leads to cystic fibrosis (CF). More than 90% of CF patients have at least one allele leading to the expression of p.F508del-CFTR. CFTR and ANO1 also mediate HCO3^−^ conductance. ANO1 expression is upregulated by the absence of CFTR and by the inflammatory cytokines in the CF airway [3]. It is generally accepted that ANO1 and BK function as ancillary Cl^−^ channels providing hydration of the residual ASL in the absence of CFTR function. Many CF patients are starting to benefit from the recently FDA-approved drugs, including correctors that increase the plasma membrane abundance of mutant CFTR and potentiators that activate the corrected CFTR channel function [6]. On-going studies examine whether modifications of the ancillary Cl^−^ channel function could help to realize the full benefit of the CFTR-based therapy. 

## 3. Biogenesis and Processing of miRNA 

microRNA (miRNA) is a class of non-coding, short single-stranded RNA playing an essential role in cellular homeostasis and disease pathogenesis by regulating gene expression. miRNAs become incorporated into a multiprotein RNA-induced silencing complex (RISC), which guides them to base-pair with the miRNA response element (MRE) in the target mRNA to mediate post-transcriptional regulation [7,8]. The miRNA genes constitute around 1%–2% of the entire human genome and encode over 2000 miRNAs, regulating one-third of all genes [9].

**Figure 1:**
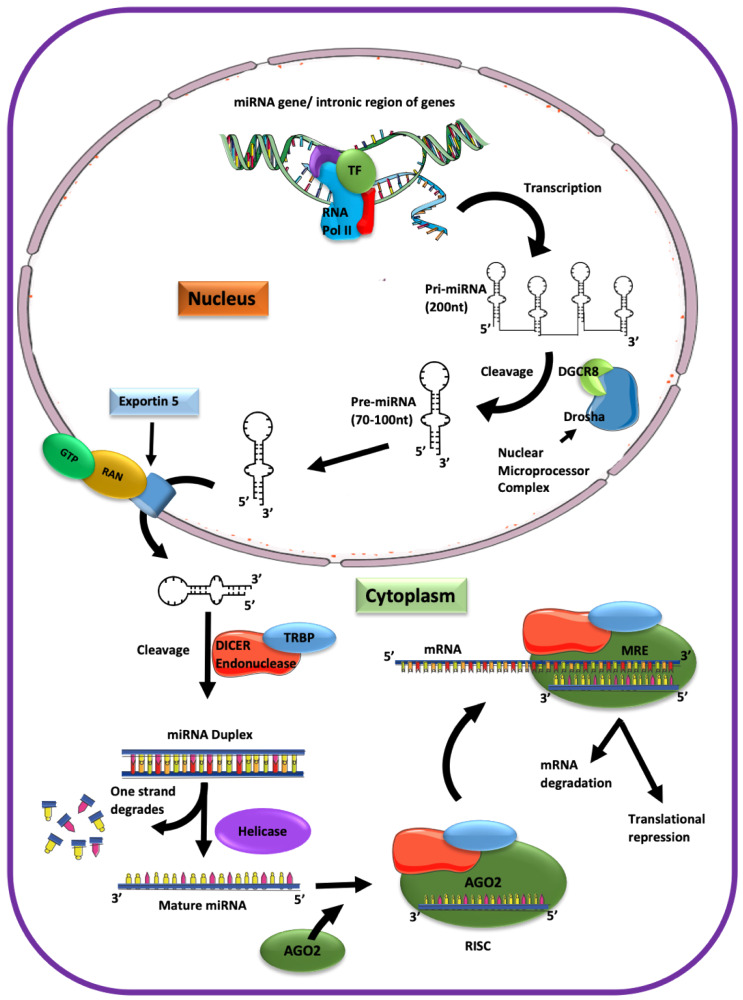
The biogenesis and processing of miRNA. Transcription of the intronic gene region yields the primary (pri)-miRNA that is targeted by the Nuclear Microprocessor Complex containing Drosha and the DiGeorge syndrome critical region gene 8 (DGCR8). The cleaved pre-miRNA is exported from the nucleus by Exportin 5. In the cytoplasm, pre-miRNA is processed by Dicer and Trans-activating response RNA-binding protein (TRBP) into 19-22 nucleotide-long miRNA duplexes. A helicase separates the two strands into a single-stranded mature miRNA recruited into the RNA-induced silencing complex (RISC) that guides the miRNA binding to the miRNA-response element (MRE), usually in the 3’ untranslated region (UTR) of the target gene.

The miRNA biogenesis starts in the nucleus and is completed in the cytoplasm (Figure 1). First, transcription of the intronic gene region with a size of approximately 200 to several thousand nucleotides yields the primary (pri)-miRNA folded into hairpin loops. The nuclear microprocessor complex containing endonuclease (type III RNase) Drosha and the DiGeorge syndrome critical region gene 8 (DGCR8) cut the pri-miRNA into 70–100 nucleotide-long precursors (pre)-miRNA [10,11,12]. Pre-miRNAs are then transported via nuclear pores into the cytoplasm by exportin 5. Next, pre-miRNA is cut into 19-22 nucleotide-long miRNA duplexes by the cytoplasmic endonucleases Dicer and the Trans-activating response RNA-binding protein (TRBP). Finally, a helicase separates the pre-miRNA duplex into a single-stranded mature miRNA that becomes incorporated into the Argonaute (Ago) containing, RNA-induced silencing complex (RISC) to exert the miRNA-mediated interference [13,14]. Although five Ago isoforms have been described, only four are associated with small non-coding RNAs in humans [15,16,17], and only Ago2 controls the miRNA function [14,15]. Ago2 facilitates the binding of miRNA to the target mRNA [15,17,18]. Subsequently, the endonuclease activity of the RNaseH-like P-element-induced wimpy testis (PIWI) domain of Ago2 cleaves the miRNA-mRNA duplex [17,18]. The Ago2-miRNA-RISC complex confers post-transcriptional repression [19]. Initial work suggested that miRNAs primarily inhibit protein translation, but the current model indicates that miRNAs also lead to degradation of the target mRNA [20].

The base-pairing of miRNA with the target mRNA is mediated by a 6–8 nucleotide-long seed sequence complementary to the MRE, usually in the 3’UTR of a target mRNA. The seed sequences start at the 2nd nucleotide and are up to the 8th nucleotide from the 5’ portion of miRNA, which participates in the MRE recognition. The thermodynamic stability and strength of miRNA–mRNA interaction, which depends on the difference in binding energy (ΔG) and AU content at the binding region, are additional factors affecting the miRNA–mRNA interaction [21]. A miRNA may have more than one seed sequence in the target mRNA. One miRNA can target one or more mRNAs involved in the regulation of more than 60% of protein-coding genes [22]. Several online tools help researchers to identify miRNA targets in silico before experimentally validating them [13,23,24,25]. 

## 4. Validation of the miRNA Role in Gene Regulation

Validation of the miRNA role in gene regulation is a complex and meticulous process. First, in silico prediction of a putative mRNA target of specific miRNA should be done using several databases providing complementary information. For example, miRBase manages the annotation of miRNAs and information about the predicted and validated target mRNAs [26]. TargetScan is a tool to predict mRNA targets and miRNAs inhibitors [27]. In vitro confirmation of the miRNA–mRNA binding can be achieved by the following assays: miRNA pull-down, Ago2 immunoprecipitation, and luciferase-based. The final confirmation of the miRNA gene regulation under specific conditions is the most challenging step. Only up to 10% of the total cellular miRNA is associated with RISC and actively participates in the miRNA-mediated interference [28,29]. Hence, the RISC-associated fraction, rather than the entire cellular level of miRNA, determines its functional pool [30]. We have recently shown that the Transforming Growth Factor (TGF)-β1 increases the total level of validated CFTR inhibitor miR-154 without increasing the RISC-associated fraction [28]. By contrast, TGF-β1 specifically recruited to RISC two other validated CFTR inhibitors, miR-143 and miR-145 [28]. The effect of miRNA on a specific mRNA target can be affected by the target mRNA expression level and the abundance of the competing mRNA targets [31]. Its binding affinity modulates the miRNA function to the mRNA target, the availability of RISC components, and the competition between different miRNAs for recruitment to RISC [32]. Furthermore, altering the expression of proteins involved in miRNA biogenesis may affect the miRNA-mediated targeting efficiency [20,33,34,35]. 

miRNAs modulate the expression of genes controlling diverse biological functions, including cellular differentiation, organogenesis, proliferation, metabolism, immune responses, and cell death programs [19,36,37,38,39,40,41,42,43,44,45]. Some miRNAs can be exported into the extracellular environment in microvesicles, serving as biomarkers for disease diagnosis or response to therapy [39,40,41,42]. Anti-miRNAs strategies may play a role as therapeutics [40,41,42,43,44]. An antisense oligonucleotide can function as a sponge binding to a specific miRNA and eliminating the downstream effects on the target genes. For example, an inhibitor of miR-145 prevented the house dust mite-induced asthma, attenuated pulmonary hyperplasia, and decreased levels of interleukins associated with allergy in the BALB/c mice lung [45]. Another approach involves an antisense oligonucleotide called target site blocker (TSB), which binds to the specific MREs in the target mRNA [43,44]. TSBs out-compete the miRNAs from interacting with specific MREs because of a higher binding affinity for mRNA [44].

**Figure 2:**
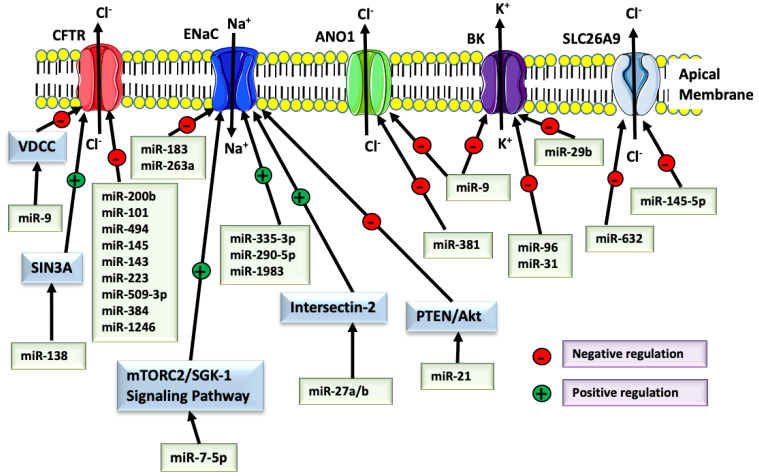
Summary of the ion transport regulation by miRNAs in human bronchial epithelial cells. miRNAs may regulate ion transport by directly targeting the channels’ mRNA or indirectly by modulating the expression of regulatory proteins and signaling pathways that control the channels’ function. CFTR: cystic fibrosis transmembrane conductance regulator; ENaC: Epithelial Na+ Channel; ANO1: Anoctamin 1; BK: the large conductance, Ca^+2^-activated and voltage-dependent K^+^ channel; SLC26A9: solute carrier family 26, member A9; VDCC: Voltage-gated calcium channel; PTEN: Phosphatase and tensin homolog; Akt: Protein kinase B (a serine/threonine-specific protein kinase); mTORC2: mammalian target of rapamycin complex 2; SGK-1: Serine/threonine-protein kinase; SIN3A: SIN3 transcription regulator family member A; miR: micro RNA.

Compelling evidence, summarized in Figure 2, demonstrates that miRNAs regulate ion transport by either directly targeting the channel’s mRNA or indirectly by modulating the expression of regulatory proteins and signaling pathways that control the channel’s function [28,46,47,48,49,50].

## 5. Role of miRNA in Regulating CFTR

The expression of the *CFTR* gene is tightly regulated in a temporal and tissue-specific manner [51,52]. Gillen et al. first reported the role of miRNAs in CFTR expression [53]. The validated CFTR inhibitors miR-101, miR-145, and miR-384 play an essential role in the switch from a strong fetal to low postnatal CFTR expression [54]. Interestingly, miR-101 negatively regulated CFTR in the adult airway cell lines but did not affect CFTR in the fetal bronchial epithelial cells. These data demonstrate that miRNAs control the temporal expression of CFTR. In the postnatal airway, the CFTR protein is abundant in the submucosal serous gland cells, much less abundant in multi-ciliated surface epithelial cells, and highly expressed in the newly identified ionocytes [55,56,57]. The role of miRNAs in controlling the cell-type-specific expression of CFTR in the airway epithelium is practically unknown.

Many miRNAs have been experimentally validated as CFTR inhibitors [50,53,54,58,59]. miR-101 and miR-494 markedly repressed CFTR expression alone and had a more substantial synergistic effect [60]. Other groups reported synergistic inhibitory effects on CFTR for the miR-145, miR-223, miR-384, miR-1246, and miR-494 or miR-509-3p together with miR-494 [50,53,58]. A reciprocal regulation was proposed that a decreased CFTR Cl^−^ channel activity may contribute to the overexpression of miR-145, miR-223, and miR-494 in the CF airway [50]. These data suggest that the severity of CF airway disease can be influenced by conditions that affect the active pools of the synergistically acting miRNAs. Enhancing the affinity of CFTR mRNA for miRNA binding is an exciting novel mechanism of CF that may explain why *CFTR* gene mutations are not identified in up to 10% CF alleles. Amato et al. reported a single nucleotide polymorphism (SNP) in the CFTR 3’UTR that increases the binding affinity of validated CFTR inhibitor miR-509-3p and reduces expression of CFTR protein, acting as a mild CFTR mutation [61]. Endale Ahanda et al. identified gene polymorphisms in the miR-99b/let-7e/miR-125a cluster that modulate the expression of these miRNAs [62]. Two of the polymorphisms in a cohort of p.F508del CF patients could modulate miRNA maturation and therefore impact the miR-99b/hsa-let-7e/hsa-miR-125a activity, acting as non-CFTR gene modifiers in CF. They may help to explain the variable severity of lung disease among CF patients with the same genotype.

The *TGF-β1* gene is a known non-CFTR modifier in p.F508del CF patients. Two SNPs present in ~40% of *F508del* homozygous patients, increase TGF-β1 protein levels, correlate with more severe lung disease, and exacerbate the damaging effects of secondhand smoke in CF patients [63,64]. Besides, *Pseudomonas aeruginosa* infection and reduced nutrition increase TGF-β1 levels in p.F508del homozygous patients [65,66,67,68]. Independent of the underlying cause, high TGF-β1 levels are strongly associated with poor outcomes [69,70,71,72,73,74]. Thus, TGF-β1 may represent a prevalent ASL inhibitor and an antagonist limiting the residual and corrected CFTR activity in CF patients. TGF-β1 inhibits CFTR mRNA level and reduces the full beneficial effects of CFTR correctors in human airway epithelial cells [75,76,77]. Although TGF-β1 is a transcriptional regulator, current data show that its inhibitory effect on CFTR is mediated post-transcriptionally via miRNAs, including miR-145 and miR-143 [28,43,59,78]. TGF-β1 changes the expression of many miRNAs, including those validated as CFTR inhibitors [28,44,78]. However, the total cellular miRNA level does not correlate with the inhibitory effect on a target gene. In agreement with this view, we have recently shown that TGF-β1 recruits specific miRNA to RISC, independently of how it affects their total cellular levels [28]. Only the miRNAs validated as CFTR inhibitors and recruited by TGF-β1 to RISC, including miR-143 and miR-145, would mediate the TGF-β1 inhibition of CFTR mRNA. This study provides another novel observation that the cellular environment of chronic lung disease, including CF, contains additional factor(s) required for the TGF-β1-mediated decay of CFTR mRNA [28]. Data showing that TGF-β1 did not inhibit CFTR mRNA in primary human airway epithelial cells from lungs without chronic disease despite recruiting miR-145 to RISC and increasing the total cellular miR-145 levels support the conclusion. These data emphasize the complexity of the TGF-β1-miRNA axis and its context-specific effects. TGF-β1 plays a significant role in the pathogenesis of other forms of lung disease, including chronic obstructive pulmonary disease (COPD), the third leading cause of death in the US, where it causes acquired CFTR dysfunction by cigarettes smoke exposure [74,79,80,81,82,83]. Environmental pollutants, including cigarette smoke, also increase TGF-β1 levels and raise the risk of sinopulmonary disease in carriers of the *CFTR* gene mutations (15,000,000 people in the US), compared to the general population [84]. The SNPs associated with high TGF-β1 levels may also contribute to the acquired CFTR dysfunction. We have shown that TGF-β1 inhibits CFTR mRNA in human bronchial epithelial cells from COPD and idiopathic pulmonary fibrosis (IPF) lungs [28]. These data suggest that miRNAs may also carry out the TGF-β1 repression in these conditions. Dutta et al. provided evidence for the role of TGF-β1 and miR-145 in cigarette smoke-induced acquired CFTR dysfunction [78]. Cigarette smoke exposure is associated with a specific signature comprised of a network of miRNAs and proinflammatory signaling cascades, leading to decreased pulmonary function [85]. Avoiding cigarette smoke exposure is the only valid measure known to date to prevent the harmful effects mediated by these miRNAs.

Some miRNAs induce CFTR expression by targeting transcriptional repressors. For example, the miR-138 mimic restored the p.F508del-CFTR expression and function by downregulating the expression of the highly conserved transcriptional repressor SIN3A [86]. Although miR-138 may have a positive effect on CFTR protein abundance and the CFTR Cl^−^ channel function, overexpression of other genes would be expected as a result of the miR-138-mediated inhibition of SIN3A. Thus, miR-138-based therapy for CF is not feasible. By contrast, blockade of the MRE in CFTR 3’UTR by TSBs can precisely restore the CFTR Cl^−^ channel activity in CF bronchial epithelial cells. De Santi et al. recently showed that TSBs directed against the miR-223-3p and miR-145-5p MREs in the CFTR 3’UTR, encapsulated in poly-lactic-co-glycolic acid (PLGA) nanoparticles and delivered to the airway in an aerosolized form, increased CFTR expression and function in CF bronchial epithelial cells [44]. Thus, TSBs emerge as potential therapeutics precisely and specifically eliminating the inhibitory effects of miRNA on CFTR, allowing the full potential of the FDA-approved CFTR modulators in the CF airway. Moreover, the prevention of the hypoxic milieu of the muco-obstructive airway disease in CF may enhance the efficacy of CFTR correctors by preventing miRNA-200b from directly targeting the CFTR mRNA [49]. 

## 6. miRNA Effects on Other Ion Channels and Transporters with a Key Role in ASL Homeostasis

ANO1 is involved in Cl^−^ and HCO3^−^ conductance, mucin production, and cytokine secretion in the airway [3,87,88,89,90,91]. Compelling data suggest that the ANO1-mediated Cl^−^ secretion is minimal under basal conditions, while it may be upregulated in conditions presenting with decreased CFTR expression or function or during inflammation [3]. One of the miRNAs upregulated in CF airway, miR-9, was found to be a negative regulator of ANO1 [92]. TSBs directed against miR-9 MRE in the ANO1 3’UTR increased the ANO1 function and mucociliary clearance in the CF airway epithelial cell models. However, the oncogenic potential of ANO1 is associated with gastric, prostate, and ovarian cancer [93]. TGF-β1 downregulates ANO1 through post-transcriptional regulation [76]. Conversely, ANO1 promotes TGF-β1 signaling in several types of cancer cells, and this effect is blocked directly by miR-381 [93]. The ubiquitous expression of ANO1 suggests the presence of tissue-specific regulation. Thus, it remains unknown whether ANO1 stimulates TGF-β1 signaling in the airway epithelial cells. It would be another reason not to upregulate ANO1 in CF. The promoter region of ANO1 contains the signal transducer and activator of transcription 6 (STAT6) binding site, leading to interleukin-4 (IL-4)-induced ANO1 up-regulation [94]. The IL-4-stimulated upregulation of ANO1 expression in the lung may be associates with asthma [95]. IL-4 level is not increased in CF patients [96]. IL-4 controls a specific miRNA signature that influences the human macrophage activation, and miR-342-3p provides a negative feedback loop, inhibiting IL-4 signaling [97]. It remains unknown whether the miRNAs controlled by IL-4 or those that inhibit IL-4 have any regulatory effects on ANO1 expression in the airway epithelium. IL-13 also activates STAT6 and is associated with allergic disease and asthma, and its expression is upregulated in CF patients [98]. miR-155 inhibits IL-13 signaling by directly targeting its receptor IL13Rα1 [99]. Interestingly, miR-155 contributes to the secretion of IL-8, a major proinflammatory mediator in the CF airway [48]. There are no published data examining how IL-13 or IL-8 signaling affects the ANO1 expression or function through miR-155. 

SCL26A9 emerges as a modulator of wild-type and mutant CFTR. Lohi et al. first characterized SLC26A9 and suggested its association with CFTR [100]. SLC26A9 mediates Cl^−^ secretion and requires functional CFTR to maintain its activity [4]. Its expression and trafficking overlap with CFTR and depend on the epithelial cell type. The SLC26A9 interactions with CFTR involve binding between its STAS (sulfate transporter and anti-sigma factor antagonist) domain and the CFTR R domain and binding between the PDZ domain with the CFTR PDZ interacting domain. The SLC26A9 function is not essential in the healthy lung but plays a critical role in preventing airway obstruction in allergic airway disease [5]. For example, SNP rs2282430 enriched in asthma patients increases SLC26A9 binding affinity to miR-632 and decreases the channel abundance [5]. Studies in mice and cultured cells showed that cigarette smoke exposure and TGF-β1 inhibit SLC26A9 via the miR-145 mediated mechanism [78]. A similar inhibitory effect was shown for CFTR [28,43]. While miR-145 is a validated CFTR inhibitor, it remains unknown whether miR-145 targets the SLC26A9 mRNA directly or indirectly by inhibiting CFTR expression. 

ENaC-mediated Na^+^ absorption depends on the Cl^−^ conductance of CFTR and plays an essential role in ASL homeostasis by increasing water reabsorption from the ASL [101,102,103,104,105]. Several miRNAs regulate ENaC expression and function. miR-21 inhibits ENaC expression via the PTEN/AKT signaling pathway [46]. A recent study in Drosophila demonstrated that miR-263a (the human ortholog of miR-183) reduced ENaC expressions while miR-183 inhibited the three subunits of human ENaC [106]. Downregulation of miR-263a in Drosophila showed a phenotypic resemblance to the CF phenotype. miRNAs can also indirectly regulate ion channels by targeting mRNA of the intermediary proteins involved in the channel biogenesis. For example, miR-7-5p inhibited mTORC2/SGK-1 signaling pathway by downregulating mRNA expression levels of both mTOR and SGK-1, leading to a subsequent reduction of ENaC expression in A549 cells [47].

By contrast, miR-335-3p, miR-290-5p, and miR-1983 increase ENaC-mediated Na^+^ transport [107]. The mechanism could be mediated by histone modification and recruitment of RNA Pol II at the enhancer locus of the *ENaC* gene or by downregulating the expression of the inhibitors of ENaC biosynthesis [107,108]. Moreover, miR-27a/b increased ENaC-mediated Na^+^ transport by inhibiting the expression of intersectin-2 that negatively regulates membrane trafficking of ENaC [109]. 

The secretion of K+ by the apical BK channel generates an electrochemical gradient for Cl^−^ secretion by CaCC and CFTR and is critical for ASL hydration [110,111,112]. Multi-ciliated airway epithelial cells are the most likely cells expressing BK channel in the airway epithelium. The pore-forming α subunit and the regulatory β subunit are encoded by the gene *Potassium Calcium-Activated Channel Subfamily M Alpha 1* (*KCNMA1)* and *Potassium Calcium-Activated Channel Subfamily M Regulatory Beta Subunit 1* (*KCNMB1)*, respectively [112,113]. The leucine-rich repeat-containing (LRRC) γ subunits play an essential regulatory role in BK channels. One α subunit and four β and γ subunits each have been identified. The association of the α subunit with different β and γ subunits modulates the properties and function of the BK channel in various tissues. Besides, there are at least ten different splice sites in the *KCNMA1* gene that diversify the channel function and membrane expression [114]. In the airway epithelium, β2 and β4 subunits and the LRRC26 γ subunit are abundant in addition to the α subunit [112]. The inflammatory mediator interferon (INF)-γ and TGF-β1 inhibited the LRRC26 mRNA level without affecting the surface abundance of the BK α subunit, leading to ASL dehydration [111,115]. These data emphasize an essential regulatory role of LRRC26 in BK channel function and ASL homeostasis. INF-γ contributes to inflammatory responses in asthma, while TGF-β1 is associated with worse outcomes in CF lung disease. From the therapeutic standpoint, it would be essential to elucidate the downstream mediators of the cytokines to inform how to design a specific blockade and rescue the LRRC26 regulatory effect on BK channel function. miR-155 is an essential mediator of inflammation by regulating members of the INF superfamily of receptors and ligands [116]. No published data examined how miR-155 affects the BK channel by regulating INF-γ signaling in airway epithelium. IL-4 inhibits the BK channel in the airway smooth muscle cells [117]. It remains unknown whether miRNAs mediate the effects.

In the adult mammalian brain, alcohol upregulates miR-9 and mediates post-transcriptional reorganization in BK mRNA splice variants by inhibiting those that contain the miR-9 MRE [118]. This mechanism contributes to alcohol tolerance. The human immunodeficiency virus (HIV) and methamphetamine affect neurotransmitter release in dopaminergic neurons by suppressing the BK splice-variants with miR-9 MRE [119]. miR-9 plays a vital role in the pathogenesis of CF airway disease, where it compromises the mucociliary clearance by directly targeting ANO1 mRNA [92]. However, the miR-9 effects on the BK channel in the airway epithelium remain unknown. Studies in other tissues show that the BK channel may be a target of other miRNAs, including miR-96 during the development of auditory hindbrain, miR-31 in ovarian cancer cells, and miR-29b in pulmonary artery smooth muscle cells [120,121,122]. The ubiquitous expression and variable role of BK channels suggest tissue- and context-specific post-transcriptional regulations. The miRNAs effects on the airway-epithelial cell-specific BK channel remain unknown.

## 7. miRNAs As Mediators of the Host–Pathogen Interactions in the Airway 

**Figure 3:**
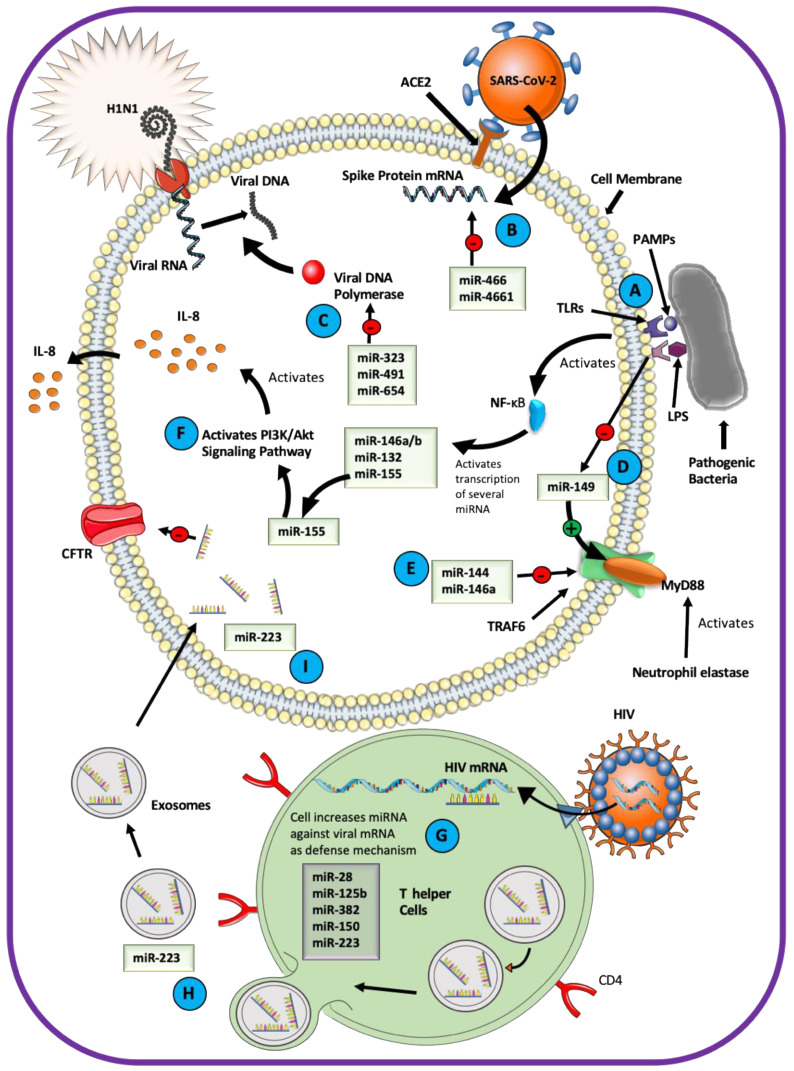
Role of miRNAs in the host–pathogen interaction. (A) Toll-like receptors (TLRs) engage with the pathogen-associated molecular patterns (PAMP) and activate the transcription factor nuclear factor-κB (NF-κB) in the host cell. (B) miRNA directly targets the mRNA of retroviruses in host cells. (C) miRNA participates in defense mechanisms and targets the genomic replication of viral DNA. (D) LPS inhibits miR-149, a direct inhibitor of MyD88, allowing activation of inflammation via the MyD88/IRAK/TRAF6 signaling pathway and expression of IL-8 in the CF airway. (E) Viral stimulated miR-144 and miR-146a target the TRAF6 signaling pathway to counteract the interferon defense responses in the human airway. (F) miRNA activated by NF-κB targets proteins that inhibit the PI3K/Akt signaling pathway to activate inflammation via IL-8. (G) Immune cells such as T helper cells can increase the expression of miRNAs against viral mRNA as defense mechanisms. (H) miRNA can also be transported from one cell to other cells through extracellular vesicles and could target ion channel proteins such as CFTR (I). ACE2: Angiotensin-converting enzyme-2; SARS-CoV-2: Severe acute respiratory syndrome coronavirus 2; PAMPs: Pathogen-associated molecular patterns; TLRs: Toll-like receptors; LPS: Lipopolysaccharide; NF-κB: nuclear factor-κB; MyD88: Myeloid differentiation primary response 88; IRAK: interleukin-1 receptor-associated kinase; TRAF6: tumor necrosis factor receptor-associated factor 6; H1N1: A subtype of Influenza A virus; HIV: Human immunodeficiency virus; miR/miRNA: micro RNA; mRNA: messenger RNA; IL-8: Interleukin-8; CFTR: Cystic fibrosis transmembrane conductance regulator; CD4: Cluster of differentiation 4; PI3K: Phosphoinositide 3-kinases; Akt: Protein kinase B (a serine/threonine-specific protein kinase).

miRNAs are essential mediators of the host–pathogen interactions in the airway epithelium (Figure 3). Pathogens change transcription of the host miRNAs after the host Toll-like receptors (TLRs) engage with the pathogen-associated molecular pattern (PAMP) and activate the transcription factor nuclear factor-κB (NF-κB) [123,124,125,126]. For example, lipopolysaccharide (LPS) functions as a PAMP and induces a variety of miRNA-mediated responses in the airway [127,128,129,130]. LPS increases the expression of miR-132, miR-146a/b, and miR-155 through TLR4 signaling [131]. Of these, miR-155 is responsible for normal B cell differentiation and antibody production, antiviral CD8+ T cell responses via INF signaling, and the proinflammatory IL-8-mediated phenotype in CF airway by activating the PI3K/Akt signaling pathway [48,116,131]. LPS inhibits miR-149, a direct inhibitor of Myeloid differentiation primary response 88 (MyD88), allowing activation of the MyD88/interleukin-1 receptor-associated kinase (IRAK)/tumor necrosis factor receptor-associated factor 6 (TRAF6) signaling pathway and expression of IL-8 in the CF airway [132,133]. LPS downregulated ENaC mRNA in rat alveolar epithelial cells and inhibited ENaC protein abundance by the miR-21/PTEN/AKT-dependent pathway [46,134]. 

miRNAs play a role in the host defenses against viral pathogens [135,136]. miR-323, miR-491, and miR-654 inhibit replication of the H1N1 Influenza A virus [137]. A study exploring the role of miRNA in antiviral immunity against HIV showed enrichment of miRNAs inhibiting HIV replication, such as miR-28, miR-125b, miR-150, miR-223, and miR-382 in T helper cells [138]. Downregulation of these miRNAs increased the viral protein translation in the T helper cells. miRNA originating in the immune cells can be packaged as cargo in exosomes and transported to other cell types. miR-223 is delivered naturally into epithelial cells through the exosomal transfer mechanism [139]. As discussed earlier, miR-223 targets the CFTR mRNA, and blocking its binding site in CFTR can increase CFTR expression and function [44]. The probability of exosomal transfer of miR-223 to airway epithelial cells explains, at least in part, the mechanism utilized by respiratory pathogens to inhibit CFTR expression and impair the ASL homeostasis. This mechanism helps to explain why viral infections compromise the efficacy of CFTR-directed therapy in CF patients. Viral pathogens can also use miRNA to reduce host survival [140,141,142,143]. For example, the influenza virus stimulates the expression of miR-144 and miR-146a in the human airway to target the TRAF6 signaling pathway counteracting the INF (type I and III) defense responses [144,145]. 

Coronaviruses (CoVs) can cause severe respiratory infection, and miRNAs play an essential role in the host–virus interaction. The severe acute respiratory syndrome (SARS)-CoV-2, associated with the CoVID-19 pandemic, is predicted to elicit a global change in the host miRNA profile and may also utilize virus-encoded miRNAs to infect the host [146,147]. The host-derived miR-466-3p and miR-4661-3p are predicted to target the SARS-CoV-2 viral spike protein that attaches to the host angiotensin-converting enzyme 2 (ACE2). The virus-encoded miR-147-3p could enhance the expression of host transmembrane serine protease 2 (TMPRSS2) utilized for viral entry into the host cell [148]. Numerous other miRNAs are predicted to target structural and functional viral proteins such as the spike, envelop, membrane, and nucleocapsid protein, as well as different open reading frames of SARS-CoV-2 [149]. 

A computational model predicts that host-encoded miRNAs may bind directly to the RNA of the Middle East Respiratory Syndrome (MERS)-CoV [150]. Of the 13 miRNAs, 10 have no validated or predicted role in human or animals, while the remaining three, miR-18a-3p, -6865-5p, and miR-342-3p, have well-described roles in human pathology. It remains to be determined how these miRNAs affect the host–virus interaction and whether they can be utilized for antiviral strategies. In a recent effort to design a therapy for SARS-CoV-2, a small interfering RNA (mode of action similar to miRNA) was shown to inhibit the expression of spike protein in the SARS-CoV-2-infected cells [151]. The nucleocapsid protein of the common-cold-associated CoV-OC43 binds the NF-κB inhibitor miR-9 and potentiates activation of the NF-κB pathway [152]. The nucleocapsid is an essential structural protein with conserved function across the CoV family, and this study may help to inform about the mechanisms used by other CoV viruses to evade the host immune system.

## 8. Can the miRNA-Based Therapy Restore ASL Homeostasis in Airway Disease?

At present, there are no approved miRNA-based therapies to restore abnormal ASL homeostasis, but strategies for a variety of chronic airway conditions and respiratory infections are being investigated [153,154]. The primary requirements for miRNA-based therapy are specificity without the off-target effects, stability, and targeted delivery. There are significant barriers to achieving these goals, illustrated by recent trials with antisense-based oligonucleotide approaches (known as antagomirs) against miR-122 for inhibition of Hepatitis C replication [155,156,157,158]. These approaches were well tolerated in vitro and in vivo. The targeted delivery of the miR-122 antagomir to hepatocytes was achieved by conjugation of the antagomir with *N*-acetylgalactosamine [159]. However, miR-122 was identified as a tumor suppressor, raising concerns about the safety of its inhibition [160,161]. The anti-miR-122 approach has been discontinued [162]. 

**Figure 4:**
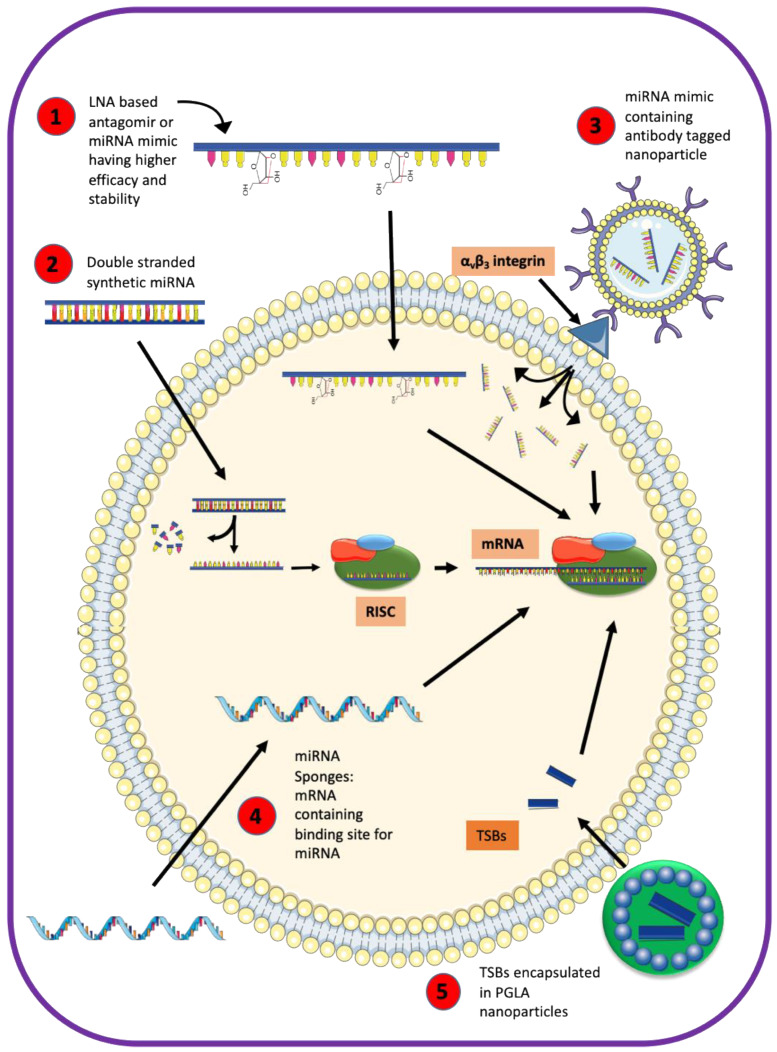
Approaches for miRNA-based therapeutic strategies. The following strategies increase stability, efficacy, and specificity of miRNA-based approaches: (1) locked nucleic acid (inaccessible RNA) modification is used in miRNA mimics or antisense-oligonucleotides (antagomirs), where the ribose moiety contains an extra bridge connecting the 2’ oxygen and 4’ carbon; (2) synthesis of double-stranded synthetic miRNA oligonucleotides; (3) coupling of miRNA mimics to antibody-coated nanoparticles; (4) delivery of mRNA expression vectors, containing miRNA sponges: mRNA containing a binding site for miRNA; and (5) using target site blockers (TSBs) encapsulated in poly-lactic-co-glycolic acid (PLGA) nanoparticles. LNA: locked nucleic acid; miRNA: micro RNA; mRNA: messenger RNA; RISC: RNA induced silencing complex; TSB: target site blockers; PLGA: poly-lactic-co-glycolic acid.

Unlike miRNA inhibitors or miRNA mimics that may affect all genes downstream of a miRNA, TSBs are specific to a particular miRNA–mRNA interaction. As a proof of principle that TSBs can serve as therapeutics to restore ASL homeostasis in CF, De Santi et al. recently showed positive effects of TSBs on the expression and function of p.F508del-CFTR in airway epithelial cells [44]. Additional studies would have to examine whether this strategy could be used in humans. Strategies addressing stability, tissue specificity and efficacy of miRNA-based therapy are summarized in Figure 4 and include encapsulating TSBs in PLGA nanoparticles, use of locked nucleic acid backbone containing miRNA, employing double-stranded synthetic miRNA oligonucleotides, a coupling of miRNA mimic to antibody-coated nanoparticles, or delivery of miRNA expression vectors [44,157,163,164,165,166,167]. Exosomes and extracellular vesicles provide an isolated environment for miRNAs and are considered useful for developing targeted therapies in respiratory diseases [168,169,170]. Liposomes have been used for packaging and delivering small molecules as therapeutics [158,171]. 

## 9. Summary

ASL homeostasis is critical for the airway integrity and host defenses. miRNAs regulate ASL by affecting the expression and function of ion channels and transporters. miRNAs serve as tools in the interactions between respiratory pathogens and the host. Understanding the complex role of miRNAs opens new horizons for designing miRNA-based therapies to restore ASL homeostasis during respiratory infection and chronic airway disease. However, significant barriers have to be overcome to deliver safe and effective miRNA-based treatment.

## Abbreviations

Ago	Argonaute
ANO1	Anoctamin-1
ASL	Airway surface liquid
BK Channel	The large conductance calcium activated and voltage dependent potassium channel
CaCC	Calcium activated chloride channel
CF	Cystic fibrosis
CFTR	Cystic fibrosis transmembrane conductance regulator
COPD	Chronic obstructive pulmonary disease
DGCR8	DiGeorge syndrome critical region gene 8
ENaC	Epithelial sodium channel
HIV	Human immunodeficiency virus
IL	Interleukin
INF	Interferon
IPF	Idiopathic pulmonary fibrosis
LPS	Lipopolysaccharide
LRRC	Leucin-rich repeat-containing
miRNA/miR	Micro RNA
MRE	miRNA response element
NF-κB	Nuclear factor κB
PAMP	Pathogen-associated molecular pattern
PLGA	Poly-lactic-co-glycolic acid
RISC	RNA induced silencing complex
SARS-CoV-2	Severe acute respiratory syndrome coronavirus 2
SLC26A9	Solute carrier family 26, member A9
SNP	Single nucleotide polymorphism
STAS	Sulphate transporter and anti-sigma factor antagonist
TGF-β1	Transforming growth factor β1
TLR	Toll-like receptor
TMEM16A	Transmembrane member 16A
TRBP	Trans-activating response RNA-binding protein
TSB	Target site blocker
UTR	Untranslated region
